# Adhesin genes and biofilm formation among pediatric *Staphylococcus aureus* isolates from implant-associated infections

**DOI:** 10.1371/journal.pone.0235115

**Published:** 2020-06-22

**Authors:** Catherine E. Foster, Melissa Kok, Anthony R. Flores, Charles G. Minard, Ruth A. Luna, Linda B. Lamberth, Sheldon L. Kaplan, Kristina G. Hulten

**Affiliations:** 1 Department of Pediatrics, Section of Infectious Diseases, Baylor College of Medicine and Texas Children’s Hospital, Houston, TX, United States of America; 2 Division of Infectious Diseases, Department of Pediatrics, Center for Antimicrobial Resistance and Microbial Genomics, McGovern Medical School, University of Texas Health Science Center at Houston, Houston, TX, United States of America; 3 Institute for Clinical and Translational Research, Baylor College of Medicine and Texas Children’s Hospital, Houston, TX, United States of America; 4 Department of Pediatrics, Section of Pathology and Immunology, Baylor College of Medicine and Texas Children’s Hospital, Houston, TX, United States of America; Thomas Jefferson University, UNITED STATES

## Abstract

**Background:**

Microbial surface component recognizing adhesive matrix molecules (MSCRAMMs) facilitate *Staphylococcus aureus* adherence to host tissue. We hypothesized that *S*. *aureus* isolates from implant-associated infections (IAIs) would differ in MSCRAMM profile and biofilm formation *in vitro* compared to skin and soft tissue infection (SSTI) isolates.

**Methods:**

Pediatric patients and their isolates were identified retrospectively. IAI and SSTI isolates were matched (1:4). Pulsed field gel electrophoresis was performed to group isolates as USA300 vs. non-USA300. Whole genome sequencing was performed and raw sequence data were interrogated for presence of MSCRAMMs (*clf*A, *clf*B, *cna*, *ebh*, *efb*, *fnbp*A, *fnbp*B, *isd*A, *isd*B, *sdr*C, *sdr*D, *sdr*E), biofilm-associated (*icaA*,*D*,*B*,*C)*, and Panton-Valentine leukocidin (*lukSF-PV*) genes, accessory gene regulator group, and multilocus sequence types. *In vitro* biofilm formation was assessed for 47 IAI and 47 SSTI isolates using a microtiter plate assay. Conditional logistic regression was performed for analysis of matched data (STATA11, College Station, TX).

**Results:**

Forty-seven IAI and 188 SSTI isolates were studied. IAI isolates were more often methicillin susceptible *S*. *aureus* and non-USA300 vs. SSTI isolates [34 (72%) vs. 79 (42%), p = 0.001 and 38 (81%) vs. 57 (30%) p <0.001, respectively]. Greater than 98% of isolates carried *clf*A, *clf*B, *efb*, *isd*A, *isd*B, and *icaA*,*D*,*B*,*C* while *cna* was more frequently found among IAI vs. SSTI isolates (p = 0.003). Most isolates were strong biofilm producers.

**Conclusions:**

*S*. *aureus* IAI isolates were significantly more likely to be MSSA and non-USA300 than SSTI isolates. Carriage of MSCRAMMs and biofilm formation did not differ significantly between isolates. Evaluation of genetic polymorphisms and gene expression profiles are needed to further delineate the role of adhesins in the pathogenesis of IAIs.

## Introduction

*Staphylococcus aureus* is a common colonizer of humans and is a ubiquitous pathogen equipped with a diverse assemblage of virulence factors. *S*. *aureus* is capable of producing a wide array of infections ranging from minor skin and soft tissue infections (SSTI) to potentially life-threatening invasive infections including medical device infections. Associations between *S*. *aureus* genotypes and site specific infections or infection severity have been reported [1–6]. Cell-wall anchored proteins are important virulence factors that are involved in many aspects of the infectious process including adhesion, biofilm formation and evasion of innate and adaptive immunity [7]. *S*. *aureus* adherence to host-tissue ligands is mediated by a family of adhesins or genetically defined microbial surface proteins referred to as microbial surface component recognizing adhesive matrix molecules (MSCRAMMs) [7]. Over twenty MSCRAMMs have been described, including fibronectin-binding proteins A (FnBpA) and B (FnBpB) which bind to fibronectin, clumping factors A (ClfA) and B (ClfB) which bind to fibrinogen, and collagen adhesin (Cna) which binds to collagen [7–9]. Historically, MSCRAMMs have been defined by their ability to adhere to the extracellular matrix of the host and display a common LPXTG motif through which they are anchored to cell wall peptidoglycan [10, 11]. Many of the host proteins to which MSCRAMMs attach remain unknown and MSCRAMMs frequently have redundant and overlapping functions. MSCRAMMs are speculated to play a role in the pathogenesis of *S*. *aureus* implant-associated infections (IAIs) by promoting colonization, invasion, and biofilm formation [7–9].

The ability of *S*. *aureus* to form biofilm contributes to the organism’s virulence and is integral to the development of indwelling medical device infections [12]. One of the most well-known mechanisms of biofilm formation is the production of a cell surface polysaccharide, polysaccharide intracellular adhesion (PIA) or poly-N-acetyl B1-6 glucosamine (PNAG), encoded by the *icaADBC* operon, though several *ica*-independent mechanisms of biofilm production have also been described [12–14]. Additional mechanisms of biofilm formation can include the release of extracellular DNA (eDNA) from lysed cells and phenol-soluble modulins (PSMs) [13–15].

IAIs are a major cause of healthcare-associated infections, including for children, with significant implications for patient morbidity as well substantial economic impacts [16, 17]. *S*. *aureus* is a common cause of IAIs, and the most frequent cause of orthopedic implant infections [15]. Data describing the molecular epidemiology of IAIs, however, are lacking, especially in children. It is unclear if *S*. *aureus* isolates causing IAIs differ from *S*. *aureus* isolates causing less complicated SSTIs in their repertoire of adhesins and biofilm-forming capabilities. A better understanding of the bacterial factors governing pathogenesis of IAIs with a focus on bacterial adherence and biofilm formation may lead to innovations or mechanisms for the prevention of pediatric IAIs.

In this study, we aimed to molecularly characterize the isolates of pediatric patients with *S*. *aureus* IAIs seen at Texas Children’s Hospital (TCH) using whole genome sequencing (WGS). We hypothesized that *S*. *aureus* isolates from IAIs would differ in genotype, MSCRAMM and biofilm gene profiles, and ability to form biofilm *in vitro* compared to SSTI isolates.

## Methods

### Population and *S*. *aureus* isolates

Patients and isolates were identified retrospectively from an ongoing *S*. *aureus* surveillance study database at TCH from January 1, 2008—June 30, 2016. This surveillance study has been in place since August 1, 2001. All *S*. *aureus* isolates are stored at -80ºC in the Dr. Edward O. Mason, Jr. Infectious Diseases Research Laboratory [2]. This study was approved by the Baylor College of Medicine Institutional Review Board. Pediatric patients (<21 years of age), evaluated at TCH were included if they had an implant or hardware in place and the infection was related to the operative procedure by fulfilling the Centers for Disease Control and Prevention (CDC)/ National Healthcare Safety Network (NHSN) criteria for a deep incisional surgical site infection (SSI) (eg involving the fascia and muscle layers) [18]. Patients with superficial incisional SSIs, exit or pin site infections, and patients with an infected tracheostomy, gastrostomy tube, ventriculoperitoneal shunt, or a peritoneal dialysis catheter were excluded. Individual charts were reviewed for operative reports, culture data, histopathology, and imaging. The clinical course and outcomes of patients with *S*. *aureus* IAIs were discussed in detail in a previous publication [19].

Patient age and date of isolation matched SSTI control isolates were selected in a 1:4 ratio of case to control isolates from the *S*. *aureus* database.

### Molecular analysis

Isolates were typed by pulsed field gel electrophoresis (PFGE) in order to group the isolates as USA300 vs nonUSA300 as previously described [2, 20]. Isolates were classified as USA300 if they differed by ≤ 4 bands from a USA300.0114 reference strain (TCH 1516) [21]. Polymerase chain reaction (PCR) to determine *agr* type was performed on isolates that were nontypeable by WGS using published primers [1].

### Sequencing and data analysis

Genomic DNA was isolated using the MoBio Ultraclean Microbial Genomic DNA Isolation Kit (Qiagen, Valencia, CA). DNA libraries were prepared for WGS using Bioo Scientific NEXTflex Rapid DNA-Sequencing kit (Austin, TX) according to the manufacturer’s guidelines. WGS was performed using a MiSeq (Illumina, San Diego, CA) platform instrument using 150-bp paired-end sequencing. *De novo* assembly of the isolates and multi-locus sequence typing (MLST) was performed using the CLC Genomics Workbench, version 10.1.1 (Qiagen). MLST sequence types were compared using eBurst analysis (www.phyloviz.net). Accessory gene regulator (*agr*) groups I-IV were assigned for each isolate using *agrD* [22]. Assembled reads derived from raw WGS data for each isolate were interrogated for the presence of MSCRAMM (*clf*A, *clf*B, *cna*, *ebh*, *efb*, *fnbp*A, *fnbp*B, *isd*A, *isd*B, *sdr*C, *sdr*D, *sdr*E) (Table 1), biofilm-associated (*icaA*, *icaB*, *icaC*, *icaD)*, and Panton-Valentine leukocidin (PVL) (*lukSF-PV*) genes using the Microbial Genomics module (version 2.5.1) of CLC Genomics Workbench. Criteria for gene presence was defined by 90% gene identity and a minimum 80% gene length. For surface protein genes with repetitive sequences (*clf*A, *clf*B, *cna*, *ebh*, *sdrC* and *sdrD*), the minimum gene length threshold was adjusted to 60%.

**Table 1:** List of selected *Staphylococcus aureus* surface proteins and genes studied.

Protein name, abbreviation	Gene symbol
Clumping factor A, ClfA	*clfA*
Clumping factor B, ClfB	*clfB*
Collagen binding protein, Cna	*cna*
Extracellular matrix-binding protein homologue, Ebh	*ebh*
Extracellular fibrinogen-binding protein, Efb	*efb*
Fibronectin binding protein A, FnBpA	*fnbpA*
Fibronectin binding protein B, FnBpB	*fnbpB*
Iron-regulated surface determinants A, IsdA	*isdA*
Iron-regulated surface determinants B, IsdB	*isdB*
Serine aspartate protein C, SdrC	*sdrC*
Serine aspartate protein D, SdrD	*sdrD*
Serine aspartate protein E, SdrE	*sdrE*

### Microtiter plate (MTP) biofilm assay and scoring system

*In vitro* biofilm formation was assessed for 47 IAI isolates and 47 SSTI isolates (the first matched control isolate per IAI) using a microtiter method [23–25]. Bacterial cultures were resuspended in normal saline and the solution diluted to achieve an optical density (OD_600_) of 0.9–1.1 which is equivalent to 10^6^ colony forming units/ml. The wells of a polystyrene 96-well microtiter plate (Falcon, Corning, NY) were inoculated with 200 μL of bacterial solution + trypticase soy broth with 1% glucose. Following 24-hour incubation at 35ºC the wells were decanted and washed three times using sterile saline phosphate buffer and dried in a hybridization oven for 1 hour at 60ºC. The wells were stained with safranin for 30 minutes and excess stain removed by washing three times with sterile water. The safranin dye bound to adherent cells was dissolved in 200μL ethanol per well and results read at OD_490_ using a microtiter plate reader (Dynex MRX Revelation). *S*. *aureus* RN4220 was used as the positive control and uninoculated media was the negative control. All isolates were tested in quadruplicates and the experiments were done three times. Biofilm formation by isolates was classified according to a modified scoring system. In the scoring system described by Stepanovic *et al*., biofilm production is quantified by establishing a cutoff OD (ODc) for each microtiter plate, which is defined as the average OD of negative control + 3 x standard deviation (SD) of negative control. Strains are then classified as: a non (OD ≤ OD_c_), weak (OD_c_< OD ≤ 2 x OD_c_), moderate (2 x OD_c_< OD ≤ 4 x OD_c_), or strong (4 x OD_c_< OD ≤ 8 x OD_c_) producer of biofilm [24]. To further stratify biofilm production, we added a classification of very strong (OD > 8 x OD_c_) producer.

### Statistical analysis

Conditional logistic regression was used for analysis of matched data using STATA11 software (College Station, TX). Two-tailed *P*-values <0.05 were considered statistically significant.

## Results

### Molecular characterization

Forty-seven IAI and 186 SSTI isolates were compared (two patients were matched at a 1:3 ratio). MLST and *agr* group (I-IV) assignments are described in Table 2. Most isolates were either ST8 or ST5. The IAI isolates displayed 19 STs without a predominant ST. SSTI isolates were predominately ST8 (68%) and belonged to 39 STs. We identified 16 new sequence types (ST4717-4721 and ST4728-4738), and 14 of these were from SSTI isolates. Overall, the most common *agr* group was group I (74%) followed by group II (15%); among the IAI isolates, 40% were *agr* group II.

**Table 2:** Molecular characteristics of *Staphylococcus aureus* isolates from implant-associated infection (IAI) isolates and skin and soft tissue infection (SSTI) isolates.

Molecular Characteristic	All isolates	IAI isolates	SSTI isolates	P
	n = 235 (%)	n = 47 (%)	n = 188 (%)	
MRSA	122 (52)	13 (28)	109 (58)	0.001
MSSA	113 (48)	34 (72)	79 (42)	
USA300^a^	139 (59)	8 (17)	131 (70)	<0.0001
*agr* group	n = 233 (%)	n = 47 (%)	n = 186 (%)	
I	173 (74)	21 (45)	152 (82)	<0.0001^b^
II	34 (15)	19 (40)	15 (8)	
III	17 (7)	6 (13)	11 (6)	
IV	8 (3)	0	8 (4)	
NT	1 (0)	1 (2)	0	
MLST	n = 233 (%)	n = 47 (%)	n = 186 (%)	
8	137 (59)	11 (23)	126 (68)	<0.0001^c^
5	19 (8)	11 (23)	8 (4)	
72	6 (3)	1 (2)	5 (3)	
30	6 (3)	3 (6)	3 (2)	
121	5 (2)	0	5 (3)	
45	5 (2)	2 (4)	3 (2)	
15	4 (2)	3 (6)	1 (1)	
188	4 (2)	3 (6)	1 (1)	
508	3 (1)	1 (2)	2 (1)	
Other (36 STs)	39 (17)	10 (21)	29 (16)	
NT	5 (2)	2 (4)	3 (2)	
*pvl* positive	143 (61)	7 (15)	136 (72)	<0.0001

NT, nontypeable

^a^One IAI isolate was not typable by pulsed-field gel electrophoresis

^**b**^Conditional logistic regression comparing *agr* group I (1) to *agr* groups II, III, and IV (0), odds ratio 0.17, 95% confidence interval 0.08–0.36

^c^Conditional logistic regression comparing sequence type (ST)8 (1) to all other STs (0), odds ratio 0.12, 95% confidence interval 0.05–0.29

**Fig 1:**
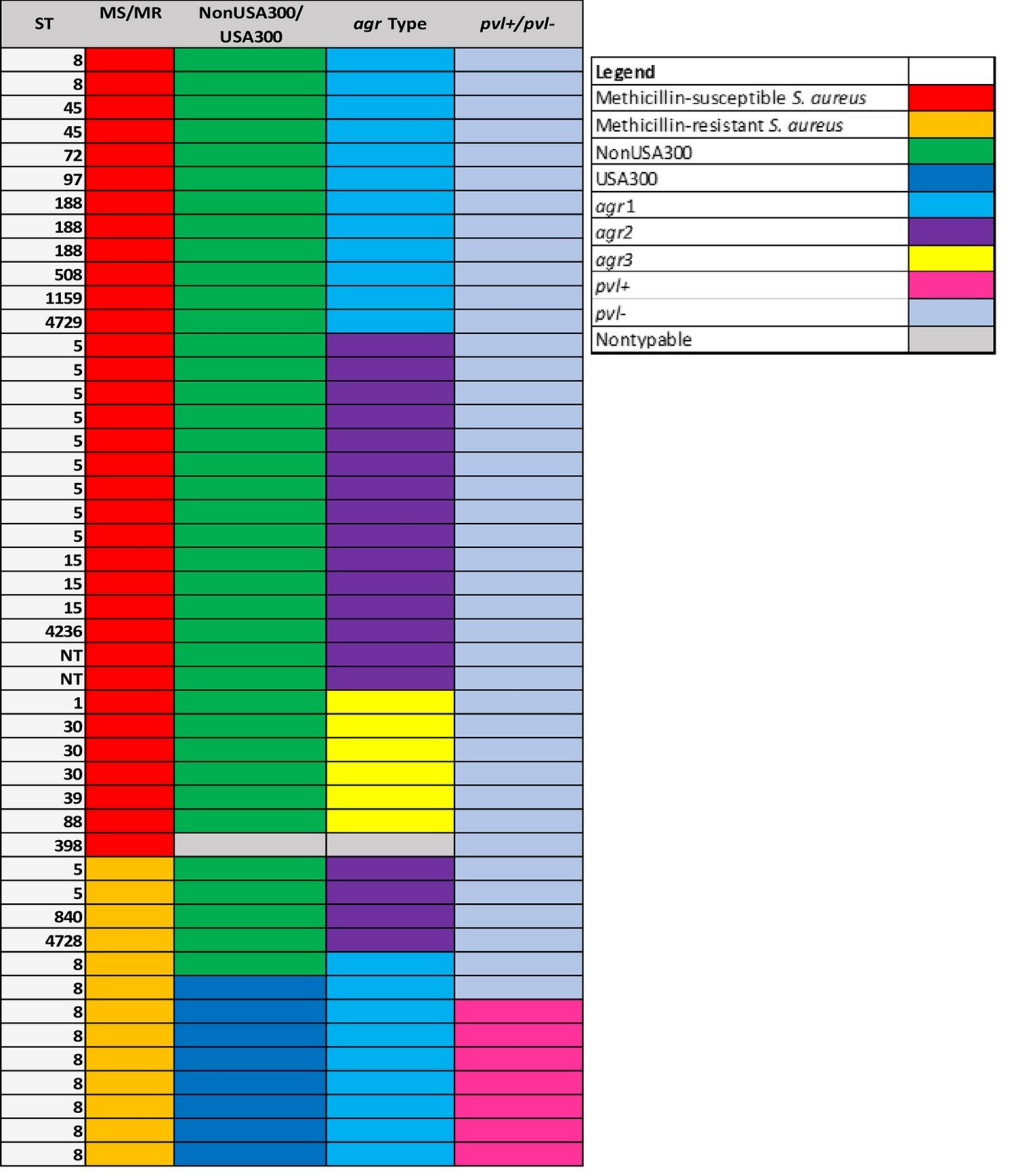
Color schematic of the forty-seven implant-associated infection (IAI) isolates.

Molecular characteristics of the 47 IAI isolates are shown in Fig 1. The majority (34/47, 72%) of IAI isolates were MSSA versus 79/188 (42%) of the SSTI isolates (OR 3.3, 95%CI 1.67–6.63, *P* = 0.001). IAI isolates were also more often non-USA300 (38/47, 81%) compared to SSTI isolates of which 57 (30%) were non-USA300 (OR 11.4, 95%CI 4.67–27.7, *P* <0.0001). Only 7 (15%) of the IAI isolates carried the *pvl* genes versus 136 (73%) of the SSTI isolates. Among MRSA isolates, 74% of 120 harbored *pvl*. In contrast, 26% of 113 MSSA carried the *pvl* genes.

The frequencies of individual MSCRAMM genes among IAI and SSTI isolates are presented in S1 Table. The majority of genes were present in all isolates with no differences observed relating to the type of infection or methicillin susceptibility. The odds of carrying *cna* was 3.9 times higher among the IAI versus SSTI isolates (95%CI 1.59–9.63, *P* = 0.003), although less than 25% of IAI isolates carried the *cna* gene For the fibronectin binding protein genes *fnbpA and fnbpB*, no significant differences were observed when comparing IAI to SSTI isolates, although a trend was observed for lesser prevalence of *fnbpB* among IAI isolates (*fnbpA*: OR 0.48, 95%CI 0.17–1.35, *P* = 0.17; *fnbpB*: OR 0.44, 95%CI 0.18–1.08, = 0.07). The biofilm-associated genes (*icaA*, *icaB*, and *icaD*) were present in all isolates and *icaC* was present among all IAI and in 99% of SSTI isolates.

### Biofilm formation

**Fig 2:**
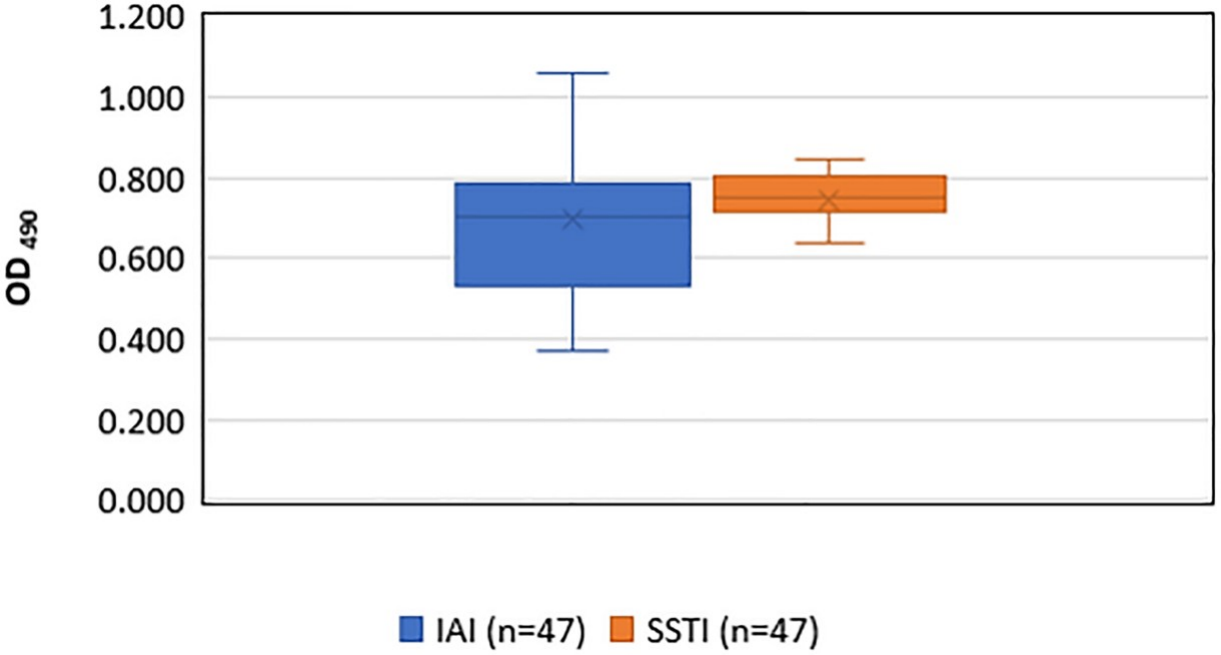
*In vitro* biofilm production among *Staphylococcus aureus* implant-associated infection (IAI) and skin and soft tissue infection (SSTI) isolates. OD, Optical density.

Biofilm formation was assessed for 47 IAI and 47 SSTI isolates. Nearly all isolates were strong or very strong biofilm producers *in vitro* (Table 3 and Fig 2). Ten IAI isolates vs 25 SSTI isolates had very strong biofilm formation. Only 3 isolates were moderate biofilm producers and there were no isolates with weak or no biofilm production. All moderate biofilm producing isolates were from IAIs and belonged to *agr* II group.

**Table 3:** Biofilm formation of pediatric *Staphylococcus aureus* isolates from 2008–2016.

Cut-off value calculation (OD_c_) ^a^	Biofilm formation	IAI isolates	SSTI isolates	P^b^
		n = 47 (%)	n = 47 (%)	
OD > 8 x OD_c_	Very strong	10 (21)	25 (53)	0.004
4 x OD_c_< OD ≤ 8 x OD_c_	Strong	34 (72)	22 (47)	
2 x OD_c_< OD ≤ 4 x OD_c_	Moderate	3 (6)	0	
OD_c_< OD ≤ 2 x OD_c_	Weak	0	0	
OD ≤ OD_c_	None	0	0	

^a^Optical density cut-off = average OD of negative control + 3 x standard deviation (SD) of negative control

^**b**^Conditional logistic regression, comparing very strong (1) to strong, moderate, and weak categories (0); odds ratio 0.12, 95% confidence interval 0.03–0.51

## Discussion

*S*. *aureus* is a pathogen capable of producing a wide spectrum of infections. While the importance of host factors in the infectious process has been widely recognized, the pathogenic potential deriving from the battery of virulence factors of different lineages of the bacterium have also been reported [1–6]. Previous findings among community-onset healthcare-associated (CO HCA) *S*. *aureus* infections in children at TCH, found CO HCA-MSSA isolates (24.1% of 357) to be more associated with invasive infections compared to CO HCA-MRSA isolates (8.9% of 542) (*P*<0.001) [2]. The USA300-ST8-agr1-*pvl+* genotype has been the predominant cause of both SSTI and invasive *S*. *aureus* infections at our institution, since the early 2000’s. While USA300 is most commonly associated with MRSA, we found a proportion (25–35%) of MSSA to also to be of this genotype [3]. We have identified associations between *pvl+* isolates and specific clinical presentations such as complicated pneumonia and venous thrombosis while other presentations, such as bacteremia, were less associated with USA300 or *pvl*+ [4–6]. In recent years, the number of infections caused by CA-MRSA has decreased as a cause of invasive CA-*S*. *aureus* infections and a recent study of invasive CA-MSSA suggested that these infections were caused by genetically diverse strains [3].

*S*. *aureus* is a common cause of IAIs, especially spinal implants or orthopedic hardware [15, 16]. To better understand genetic variation of *S*. *aureus* isolates causing IAIs in children, we investigated the presence of adhesin and biofilm-formation genes among isolates of pediatric patients with *S*. *aureus* IAIs and compared these to matched controls of *S*. *aureus* isolated from SSTIs. IAI isolates were more likely to be MSSA (72% versus 42%) and non-USA300 (81% versus 30%) when compared to SSTI isolates. Among IAI isolates only 7 carried *pvl* and all of the MSSA were *pvl*- suggesting no relevance of this toxin in implant-associated infections. MSSA is a major cause of *S*. *aureus* IAIs in both adult and pediatric patients [26, 27].

*S*. *aureus* isolates causing IAIs and SSTIs in children were genetically diverse with 45 STs identified overall. However, most SSTI isolates were ST8 (68%) and *agr* group I (82%) while, among IAI isolates, ST5 and ST8 were equally common (23% each) and *agr* group I and *agr* group II were identified for 45% and 40%, respectively. Five (3%) SSTI isolates were ST121 and *agr* group IV, a ST associated with scalded skin syndrome and recently identified as increasingly causing disease within our patient population [28]. The notion that no specific genotype was associated with device related infections suggests that the presence of implanted material alone and vulnerability of the host could outweigh differences in the pathogenicity potential between different *S*. *aureus* strains. Indeed, previously considered non-pathogenic *Staphylococcus* species, particularly coagulase-negative staphylococci, are significant pathogens in implant infections [29].

Few studies have investigated the profile of MSCRAMM and biofilm formation genes among *S*. *aureus* IAI isolates in children. We found the MSCRAMM encoding genes and the *ica-*operon to be conserved across diverse genotypes regardless of isolate origin (IAI versus SSTI). Studies of isolates from other sources showed similar findings. Ghasemian *et al*. used PCR to detect genes encoding select MSCRAMMs using 22 *S*. *aureus* isolates from hospitalized children with “systemic infections” in Iran. In their study, all isolates harbored *clfA* and *clfB*, while detection of other MSCRAMMs varied from 0–82%; no differences between MRSA and MSSA were detected [30]. McCarthy and Lindsay analyzed 58 whole genome sequences from 15 *S*. *aureus* lineages available in the public domain for variations within 25 *S*. *aureus* surface proteins. Fifty-four of the sequences were from human isolates (4 were avian, bovine, or porcine) including 17 isolates from an infection. For 26 of the sequences the infection status was publicly unavailable. Eight genes (including *fnbpA* and *isdB*) were present in all isolates and 16 genes (including *clfA*, *clfB*, *fnbpB*, *isdB*, *sdrC*, *and sdrD*) were found in nearly all *S*. *aureus* genomes [31].This study found that while MSCRAMM genes were present across diverse lineages, domain variants were observed. These variants could be associated with differences in protein expression and folding, which could be important to the organisms’ capacity for adherence to the host cell.

A 2005 study of 191 clinical *S*. *aureus* isolates from orthopedic infections, 127 of which were device related, investigated the presence of *cna*, *fnbp*A and *fnbp*B by PCR. This study found *cna* in only 46% of isolates while nearly all isolates carried *fnbpA* (98.4%) or *fnbpB* (99.5%) [32]. The authors concluded that while fibronectin-adhesins appeared to play a critical role in bone and joint implant associated infections, *Cna* was of lesser importance. In our study, *cna* was identified from only 23% of IAI isolates and 7.5% of SSTI isolates further questioning the role of Cna in SSTI or IAIs. This finding also reflects that a large number of the IAI isolates in our study were MSSA (only 22% of MSSA harbored *cna*) and the fact that USA300 isolates lack *cna* (70% of SSTI were USA300). In our study the majority of *cna*+ isolates were non-USA300 MSSA.

Overall, we and others have found genes encoding MSCRAMMs to be common across *S*. *aureus* isolates of diverse genetic backgrounds regardless of the source. Therefore, other factors, such as the local tissue changes related to the presence of an implant and the patient’s immunocompetence likely play important roles in enabling *S*. *aureus* to establish a device associated infection rather than the strain type or specific adhesin(s) and/or virulence determinants.

The *in vitro* biofilm assay used allowed only for biofilm quantification, without the ability to evaluate the mechanism of biofilm formation and was performed to investigate whether any differences could be discerned between isolates from IAIs vs. SSTIs. We hypothesized that the device related isolates would have greater biofilm production, as this phenomenon would allow for adherence to the material. We found *S*. *aureus* isolates from both IAIs and SSTIs were equally strong producers of biofilm suggesting that biofilm production is also important for the establishment of SSTIs. Other studies have found differences between isolates using the same methods and it is possible that we could have observed a broader range of biofilm formation had we included a comparator group of carriage isolates from healthy individuals [14, 30].

There are several limitations to our study. First, the isolates came from a single pediatric institution and the results may not be representative of other geographic areas or populations. As *S*. *aureus* IAIs were identified using the *S*. *aureus* surveillance database, we cannot provide a measure of overall incidence of IAIs at TCH. Secondly, the mechanisms governing MSCRAMM expression and biofilm formation are highly complex and for this study we focused only on the presence or absence of select adhesin genes. Future analyses using the WGS data will focus on defining MSCRAMM domain variations and allelic variants. Studies of the expression of these molecules could further shed light on their role in device related infections. Finally, we used a previously described *in vitro* biofilm assay to quantify biofilm production among IAI and SSTI isolates. It is possible that the use of different growth conditions, for example supplementation with glucose vs sodium chloride, or the use of another binding material could have altered the production of biofilm. Furthermore, a comparator group of colonizing isolates from healthy individuals may have provided insights into the role of biofilm formation in colonization versus disease.

In summary, *S*. *aureus* IAI isolates were significantly more likely to be MSSA and non-USA300 compared to SSTI isolates. Carriage of specific MSCRAMMs or biofilm-associated genes did not differ significantly between IAI and SSTI *S*. *aureus* isolates. While further studies evaluating the complex interactions between MSCRAMMs and biofilm formation in the pathogenesis of *S*. *aureus* IAIs are needed, the role of host and technical factors related to the operative implantation of devices remain critically important areas for investigation.

## Supporting information

S1 Table Presence of adhesin genes among implant-associated infection (IAI) isolates and skin and soft tissue infection (SSTI) isolates. (DOCX)
