# Supplementary material for: Adhesin genes and biofilm formation among pediatric Staphylococcus aureus isolates from implant-associated infections
Source: PLoS One. 2020 Jun 22;15(6):e0235115. doi: 10.1371/journal.pone.0235115 (PMC7307771; doi:10.1371/journal.pone.0235115)
Supplement: S1 Table — (DOCX) [file pone.0235115.s001.docx]

| **Gene** | **IAI**  **n=47** | **SSTI**  **n=186** | **MSSA**  **n=113** | **MRSA**  **n=120** | **Non-USA300 n=95** | **USA300**  **n=137** |
| --- | --- | --- | --- | --- | --- | --- |
| *clfA* | 98% | 99% | 99% | 98% | 97% | 100% |
| *clfB* | 98% | 99% | 98% | 99% | 98% | 99% |
| *cna* | 23% | 8% | 22% | 0% | 25% | 0% |
| *ebh* | 91% | 94% | 89% | 97% | 88% | 96% |
| *efb* | 100% | 100% | 100% | 100% | 100% | 100% |
| *fnbpA* | 87% | 94% | 85% | 99% | 83% | 99% |
| *fnbpB* | 81% | 90% | 77% | 99% | 74% | 99% |
| *isdA* | 98% | 100% | 100% | 99% | 99% | 100% |
| *isdB* | 100% | 100% | 100% | 100% | 100% | 100% |
| *sdrC* | 81% | 87% | 72% | 99% | 68% | 99% |
| *sdrD* | 87% | 91% | 88% | 93% | 79% | 99% |
| *sdrE* | 77% | 83% | 65% | 97% | 60% | 96% |
